# A triple helix model for the diffusion of Industry 4.0 technologies in firms in the Marche Region

**DOI:** 10.12688/openreseurope.15706.1

**Published:** 2023-06-09

**Authors:** Niccolò Testi

**Affiliations:** 1 Department of Law, University of Macerata, Macerata, Marche, 62100, Italy

**Keywords:** Industry 4.0, blockchain, triple helix, innovation, region, knowledge, collaboration, intermediaries

## Abstract

Background: Firms in the Marche Region, Italy, seem to struggle with understanding the importance of Industry 4.0 technologies, including blockchain technology, and delay the adoption of these innovative technologies.

Methods: This paper is based on findings of three levels of qualitative analysis: the first one is a literature review; the second uses secondary sources about the diffusion of Industry 4.0 in the Marche Region and the local institutions and policies favouring it, retrieved from institutional websites and web searches; the third uses primary data which has been collected through an explorative survey conducted by sending a questionnaire to local innovative firms between 25th and the 27th of October 2022.

Results: The literature review shows that collaboration between triple helix actors can foster innovation in firms. Secondary data about firms in the Marche Region describes an economy made prevalently of micro enterprises not capable of adopting Industry 4.0 technologies, and individuates some institutions located in the region having the role of innovation intermediaries that help firms innovate. Among the secondary sources, the new Smart Specialisation Strategy 2021-2027 of the Marche Region emphasises the role of Industry 4.0 for economic development which requires the involvement of the research and innovation actors present in the region. The survey conducted for this study shows that the firms that adopted Industry 4.0 technologies have strong ties and collaboration with triple helix institutions.

Conclusions: Based on the findings, a triple helix model is proposed to foster the diffusion of Industry 4.0 technologies in the Marche Region, where innovation intermediaries are called to manage knowledge flows both among them and between academia, government, and industry, to activate a virtuous cycle of innovation adoption and valorisation.

## Plain language summary

Industry 4.0 (I4.0) technologies can help firms innovate their products and processes, but firms in the Marche Region, Italy, struggle adopting them. To understand what policy measures are necessary to help these firms innovate, an analysis of the Marche Region’s context, key institutions, and policies is conducted and coupled with an explorative survey to firms in the Marche Region that already use I4.0 technologies. The results show that these firms collaborate with many public and private actors at the regional, national, and international levels. The paper suggests that innovation intermediaries, that are present in the region and already help firms innovate, could manage the knowledge flows among them and between government, academia, and industry so that firms can receive proper support in adopting I4.0.

## Introduction

In recent years, several firms have embarked on a trend towards automation and data exchange in their business processes, known as Industry 4.0 (I4.0) ^1^. Among the I4.0 technologies ^2^, blockchain technology (BCT) has recently emerged ^3^ as a useful tool for firms to ensure transparency and trust in data management ^4^.

From a general standpoint, I4.0 technologies enable firms to adopt new business models ^5^ and gain competitive advantages ^6, 7^. However, many firms, especially small and medium-sized enterprises (SMEs), have difficulties understanding and using innovative I4.0 technologies ^8, 9^. A triple helix (TH) approach consisting of the collaboration between government, academia, and firms in a specific territory ^10^ can help with the diffusion of innovative I4.0 technologies among firms ^11, 12^.

The objective of this study is to suggest measures to foster the diffusion of I4.0 technologies, including BCT, among firms in the Marche Region, in Italy, where many firms struggle to understand and implement I4.0 technologies ^13^. The regional context is of interest since the regional government is engaged in fostering the diffusion of innovative technologies in firms of its territory and supports it concretely with tenders ^14^. Particularly, BCT has recently drawn the region’s attention and has been addressed in both its technological road map ^15^ and Smart Specialisation Strategy (S3) ^16^. Indeed, this study is part of an innovative PhD programme created by the University of Macerata, in Italy, and promoted by the Marche Region to strengthen the relationship between academic research and the local firms’ needs, as part of the POR FSE 2014/2020 Axis 1- P.I. 8.1- R.A. 8.5 ^17^ which is financed with European funds.

The measures suggested in this paper for the diffusion of I4.0 in firms in the Marche Region leverage collaborations between existing local TH actors and other innovation intermediaries that were individuated through an analysis of the regional context. Moreover, an explorative survey of local innovative firms that are part of an exemplary innovation cluster is conducted to strengthen the findings.

This paper is structured as follows. The second paragraph presents the theoretical framework of I4.0 and its knowledge and use among firms in the Marche Region, with a focus on BCT. A TH theory applied to the diffusion of I4.0 in firms in the Marche Region is proposed. The third paragraph presents the methods. The fourth reports the findings of the analysis of the Marche Region context and institutions concerning I4.0 and the firms' answers to the survey. In the last paragraph, the results of the study are discussed, measures for the diffusion of I4.0 technologies, including BCT, among local firms are proposed, and suggestions for future research are given.

## Theoretical framework

### I4.0 and blockchain technologies

The term ‘Industry 4.0’ was first proposed in 2014 referring to the fourth industrial revolution ^1^, enabled by a group of technologies that interconnect machines and equipment through the internet into automatic and intelligent networks ^9^. I4.0 technologies include big data analytics, augmented reality, simulations, collaborative robots, 3D printing, horizontal and vertical integration, the Internet of Things (IoT), cloud storage and computing, and cybersecurity ^7^. I4.0 technologies enable firms, especially SMEs, to adopt new business models ^5^ and gain competitive advantages ^6, 7^. Factors that limit the diffusion of I4.0 in firms are mainly related to the small size of the firms ^8, 9^ and the lack of finances and specialized support in obtaining new technologies ^18^, technical and digital knowledge ^7^, and non-technical competencies ^19^.

Among the I4.0 technologies, blockchain technology (BCT) has recently emerged ^3^. A blockchain is a distributed database allowing peer-to-peer data sharing without the need for either intermediaries or trusted third parties to ensure data exchange and integrity ^20^. This is possible because the data stored in a blockchain are tamper-proof, so nobody can modify or eliminate them, and are visible to all stakeholders ^21^. Initially developed in 2008 to enable peer-to-peer transactions with the Bitcoin cryptocurrency, BCT has recently been used in many sectors as a means to make any kind of data immutable and visible to stakeholders ^22^. Industrial applications of BCT aim at bringing transparency to business processes ^23^ such as supply chain management ^24^, product traceability ^25^, accounting ^26^, manufacturing ^27^, and marketing ^28^. Firms can store the data about their processes on a blockchain to make them tamper-proof and visible to interested parties, thus enabling data transparency ^23^ which helps firms reduce information asymmetries with stakeholders ^29^ and build trust with them ^30^. BCT is transversal to all the other I4.0 technologies since it can be used together with each one of them ^31, 32^. However, firms’ digitalisation is a prerequisite for the adoption of BCT ^33^, missing which the adoption is hindered ^34, 35^. Moreover, the lack of clear regulations on BCT might discourage firms from using it ^36, 37^. Finally, BCT is still not well known to firms: Caldarelli *et al.* (2021) ^38^ stated that training by an expert consultant is crucial for the successful adoption of BCT in firms, while an Italian provider of BCT services interviewed by Compagnucci *et al.* (2022) ^39^ declared that firms, especially SMEs, need support in understanding which kind of BCT solution to adopt.

### TH and innovation intermediaries for the diffusion of I4.0 and BCT in firms

The purpose of this study is to suggest measures for the diffusion of I4.0 technologies, including BCT, in firms located in the Marche Region. Collaboration can be a source of value co-creation in an I4.0 context ^40^ and encourages firms to adopt I4.0 business models ^11^. The TH explains the positive effect of collaboration between academia, government, and firms in the economic development of territories ^10^. Academia generates basic scientific knowledge for industrial innovation ^41^ and can help firms in their technological transformation also by training skilled managerial figures who have a strong effect on the rate of diffusion of digital technologies ^42^. Governments play a key role by funding universities ^43^ and making policies that support the adoption of I4.0 in firms ^44^, whereas a lack of governmental support can hinder it ^45^. On BCT specifically, Compagnucci *et al.* (2022) ^39^ suggest that national and regional institutions should support the adoption of BCT solutions through financial and organizational measures, promoting both the tools used to favour collaboration between firms, academia, and other institutions and those adopted to support the implementation of innovation.

Within a regional TH, some intermediaries can facilitate the flow of knowledge and innovation. These are innovation intermediaries whose basic functions include process coordination and matchmaking between innovation seekers and potential solution providers, knowledge and finance brokering, testing ^46^. They facilitate the exchange and the building of new knowledge, create opportunities for experimentation, help forming partnerships of private and public actors around common goals ^47^. Since innovation intermediaries increase knowledge and resource flows amongst TH institutions and the rest of the civil society ^48^, a growing number of innovation policies rely on publicly-funded innovation intermediaries to provide knowledge-intensive services to firms, particularly SMEs ^49^.

## Methods

### Ethical statement

The study was conducted following the Horizon Europe ethics-self-assessment regarding ‘personal data’ defined as ‘information relating to an identified or identifiable natural person’ ^50^ regulated by the EU Regulation 2016/679 (GDPR) ^51^. No ethics issues were identified regarding personal data since the survey conducted for this study collected anonymised data which ‘has been rendered anonymous in such a way that the data subject can no longer be identified’ and considering that ‘completely anonymised data do not fall under the data protection rules’ ^50^. Moreover, ‘the need to ensure participants' free informed consent (with particular attention to vulnerable categories of individuals such as children, patients, discriminated people, minorities, persons unable to give consent, etc.)’ ^50^ was respected in this study since information about the study was provided to the participants, who were not vulnerable people, and written informed consent was asked and obtained from them prior to their participation in the survey as seen in section one in the questionnaire ^52, 53^ and in column B ‘Consent to participate in the study’ of the participants’ answers ^54, 55^.

### Study design

This study uses triangulation of data ^56^ by combining different sources, specifically second-hand qualitative data from a literature review and retrieved from the Web, and first-hand data from an explorative qualitative survey.

A literature review was conducted to collect evidence on the relationship between the diffusion of I4.0 and TH. A search on Scopus conducted in September 2022 using the keywords ‘Industry 4.0’ and ‘triple helix’ in the title, abstract, and keywords fields of peer-reviewed research papers. The search string used was the following: TITLE-ABS-KEY ( "industry 4.0" AND "triple helix" ) AND ( LIMIT-TO ( DOCTYPE , "ar" ) ). All years of publication were included to increase the inclusivity of results. Articles written in English were selected to avoid comprehension issues and increase the replicability of results by the international research community. The search gave only 13 results, showing that evidence on the topic of I4.0 and TH is scarce. All the articles retrieved are recent and demonstrate a growing interest in the relationship between I4.0 and TH: one was published in 2018, one in 2020, two in 2021, and nine in 2022. The abstracts and, when possible, the content of the papers were read to search for recurring themes. All the papers state that TH can help with the implementation and diffusion of I4.0. Table 1 shows the results highlighting the role attributed to the TH concerning I4.0.

**Table 1.** The scientific literature on the relationship between TH and I4.0. The table shows the results of a literature review conducted on Scopus in January 2023 using the keywords ‘Industry 4.0’ and ‘triple helix’ in the title, abstract, and keywords fields of peer-reviewed research papers. The table highlights, for each article retrieved, its methodology and the role of Triple Helix (TH) for the Industry 4.0 (I4.0) paradigm it describes.

Author(s) and date of publication	Methodology	Role of the TH for I4.0
(Reischauer, 2018) ^12^	Conceptual	Objective of policy-driven innovation discourse around I4.0.
(Steenkamp, 2020) ^65^	Conceptual	Create entrepreneurial leadership for innovation.
(Capetillo *et al.*, 2021) ^66^	Case study	Evolving into a Penta Helix to foster the diffusion of I4.0 in firms.
(Majumdar *et al.*, 2021) ^67^	Survey	Help overcome barriers to the implementation of I4.0 technologies in firms.
(AlMalki and Durugbo, 2022) ^68^	Interviews	Promote and enhance the co-evolution of institutions with technological I4.0 advances.
(Carayannis *et al.*, 2022) ^69^	Conceptual	Offer references on how knowledge and innovation could proceed in co-evolution in the context of a knowledge economy.
(Costa *et al.*, 2022) ^70^	Conceptual	Help develop teaching-learning processes which use I4.0 technologies.
(Cucculelli *et al.*, 2022) ^11^	Survey	Counterbalance the lower propensity of family managers to adopt I4.0 business models.
(Khan *et al.*, 2022) ^40^	Case study	Increase collaborative capabilities in an I4.0 ecosystem context.
(Lepore *et al.*, 2022) ^71^	Case study	Enable innovation ecosystems for developing I4.0 solutions.
(Liu and Zhu, 2022) ^72^	Case study	Lead to a knowledge spillover effect in the field of I4.0 smart factories.
(Ojubanire *et al.*, 2022) ^73^	Conceptual	Foster industrial I4.0 transformation.
(Tataj *et al.*, 2022) ^74^	Case study	Help understand key success drivers that enable science parks to deliver outstanding results in I4.0.

Following a qualitative approach, the context of the Marche Region was analysed concerning the diffusion of I4.0 technologies, including BCT, among firms of the region, and policies addressing firms’ innovation, by retrieving information from secondary sources and subjectively selecting the kind of information considered relevant for the topics addressed in terms of I4.0 and collaborations linked to the TH. Institutions and policies relevant to the diffusion of I4.0, such as the Marche Region’s S3 for the two last programming periods ^16, 57^, and tenders ^14^ were individuated by accessing the Marche Region’s institutional website ^58^ and the Marche Innovazione website ^59^ on the 8 ^th^ of October 2022. The Marche Innovazione website is the regional portal for disseminating and developing strategies for intelligent, sustainable, and inclusive economic growth in the region. It includes sources to the regional S3 and tenders of the two programming periods divided by research and development (R&D), investments, and internationalization. Additionally, web searches were conducted using the keywords ‘Industria 4.0 Regione Marche’ between the 8 ^th^ and 12 ^th^ of October 2022, which allowed to recover institutional reports regarding the digital transformation of firms in the Marche Region ^60– 63^.

An explorative qualitative survey was sent on the 25 ^th^ of October 2022 by sending an anonymous semi-structured questionnaire ^52, 53^ to all the firms with an active membership of the Fondazione Cluster Marche ^64^, which is a Foundation representing the Technology Clusters in the Marche Region. The Foundation’s members were contacted for the survey since they are innovative firms located in the Marche Region that know or use I4.0 technologies and are valuable sources of information on the implementation of I4.0 technologies in business processes.

The questionnaire was built following a continuous feedback process with two experts in qualitative research methods, an expert in I4.0 technologies, and the representatives of the Fondazione Cluster Marche who gave the final approval to the questionnaire’s content before sending it. The questionnaire was not piloted prior to this study. The questionnaire was in Italian language because it was addressed to native Italian speakers.

The questionnaire was sent by a representative of the Fondazione Cluster Marche via e-mail and was self-administered as the respondents filled it in themselves ^75^. Of the 149 firms to which the questionnaire was sent, seven compiled it, between the 25 ^th^ and the 27 ^th^ of October 2022.

The questionnaire collected the following qualitative data ^54, 55^. The first section collected generic information about the firms, such as the size and economic activity classification. The second asked the respondents to state what I4.0 technologies were used in the firms and their knowledge and use of BCT. The third assessed the collaborations that the firms have or had concerning I4.0 technologies, dividing the collaborations per type and territorial level of collaboration partners. The fourth and final section addressed the respondents’ perceived usefulness of some measures for the diffusion of I4.0 in firms in the Marche Region, leaving open questions for them to suggest initiatives additional to those indicated.

## Results

### Marche Region’s context, key institutions, and policies

The Marche Region’s economy is mostly based on clusters of SMEs ^76^. Cappelli (2020) ^77^ noted that these firms specialised in the classic industries of the Made in Italy, for example, those of timber, furniture, leather, footwear, and household appliances. However, in these industries, the technological advantage gained does not always translate into an economic advantage due to the lack of complementary skills and assets. Indeed, many firms in the Marche Region struggle to understand and implement I4.0 technologies ^13^. A 2021 report of the Osservatorio Impresa 4.0 ^61^ found that the firms in the Marche Region show significant delays in the adoption of I4.0 technologies due to their limited size. Indeed, they are mainly micro-sized enterprises (94%), followed by small and medium (5.7%), and large (0.1%) ^62^. Moreover, the report states that the delay is also caused by the lack of specific technical skills in firms and the lack of collaborations with developers of new technologies. Another factor causing the delay is the firms’ low level of digital knowledge and skills ^78^. Regarding BCT in particular, the firms’ size seems to influence the level of knowledge on BCT as it is for other I4.0 technologies. A survey conducted in 2019 revealed that 80% of SMEs did not know about BCT, 16% knew it superficially, and only 4% understood it deeply, whereas larger firms showed higher levels of awareness and deep knowledge ^22^. The lack of awareness of BCT among Italian firms was confirmed in the same year by a survey from the Italian Ministry for Economic Development ^79^. A more recent survey by Bracci *et al.* (2022) ^80^ found that Italian SMEs are quite aware of the existence of BCT but their level of knowledge is limited and the adoption rate is very low.

In the context of the region, some key institutions may help to increase the adoption of I4.0 technologies, including BCT, among firms. The authors of the 2021 report by the Osservatorio Impresa 4.0 ^61^ state that a synergistic and complementary relationship between Punto Impresa Digitale (PID) ^60^, Digital Innovation Hubs (DIHs), and Competence Centers can be leveraged in the Marche Region to help firms achieve a higher level of digitalisation and usage of I4.0 technologies. PID are an initiative of the Italian trade unions Camera di Commercio and Unioncamere offering a series of services and opportunities for firms such as basic courses on I4.0 and specific training, consultancy, or direct assistance to support digitization. DIHs are knowledge brokers that support firms and connect them with public and private actors such as universities, research centres, service providers, and corporations ^81^. Competence Centers are public-private partnerships that were created by the Italian government to carry out guidance and training activities for firms on I4.0 as well as support them in the implementation of innovation, industrial research, and experimental development projects through I4.0 technologies ^63^. Another institution which was not mentioned in the report but could have a role in the diffusion of I4.0 in the Marche Region is the Fondazione Cluster Marche ^64^, which represents the Technology Clusters in the Marche Region. Technology Clusters are aggregations of companies, universities, and research institutes that work together to promote excellence in research and innovation. The purpose of the Fondazione Cluster Marche is to enhance the capabilities of the Marche Region innovation system through the development of collaborative research and technology transfer activities. The Fondazione Cluster Marche is one of the partners of i-Labs ^82^, a laboratory which represents the physical centre of the regional Collaborative Platform on I4.0. Inside the laboratory, researchers and entrepreneurs develop, apply, and share solutions useful for improving production systems, to ensure rapid evolution towards I4.0. The i-Labs offers orientation and consultancy activities, research and development, training for companies towards I4.0 technologies, including BCT, and help firms in participating in national and regional tenders. These services are offered in cooperation with DIHs, Competence Centres, academia, and technology providers.

As for the policies, an important role in the diffusion of I4.0 in firms in the Marche Region could be played by the regional Smart Specialisation Strategy (S3) ^83^, which aims to invest European community funds to build comparative advantages and sustainable growth in the long term by using the existing territorial resources and production capacities ^84^. The first S3 plan of the Marche Region for the period 2014–2020 ^57^ contributed to an increase in the propensity of regional companies to invest in R&D activities, innovate, collaborate with the academia, develop R&D and/or activities for innovation, and increase the number of placements of highly qualified personnel. Compared with the first S3 plan, the new regional S3 plan 2021–2027 ^16^ emphasises the role of I4.0 for economic development and notes the delay in firms in the Marche Region in the adoption of these innovative technologies. Nevertheless, the new plan states that the effective implementation of the S3 requires the involvement of the research and innovation actors present in the Marche Region, for which a TH approach could be beneficial.

In the process of entrepreneurial discovery, the region has organized meetings with the stakeholders of the TH to identify needs and innovation trajectories. In particular, concerning the S3 for 2021–2027, blockchain is presented among the innovative trajectories identified. Indeed, new needs have emerged concerning new approaches based on technologies for authenticity, management, tracking and traceability also from a BCT perspective. The technology is presented as linked to competencies regarding digital technologies and engineering while the main market driver of this technology is related to inclusion and social innovation. Finally, the Marche Region expressed its interest in BCT with Regional Law number 36 of 2020 ^85^, which states that the region promotes the use of a multifunctional IT platform based on BCT for registering and managing funds and tenders, tracing the typical products of firms of the Marche territory, and rewarding citizens for their participation to public endeavours.

### Survey results

In order to provide an empirical basis for the measures suggested in this paper for the diffusion of I4.0 technologies in local firms, a questionnaire was sent to the 149 firms that are members of the Fondazione Cluster Marche, to which seven of them responded (Testi, 2023a) ^54, 55^. Four of them are large-sized, one is medium, and two are small. The recently updated ATECO 2007 classification of economic activities by Istat ^86^ was used to classify the firms by sector. Four are manufacturers, one is in the sector of agriculture, forestry, and fishing, one offers services to firms, and one conducts professional, scientific, and technical activities ( Table 2).

**Table 2.** Size and ATECO sector. The table shows the size and sector of the firms that participated to the survey, numbered from 1 to 7.

Firm n.	Size	Sector (ATECO 2007)
1	Large	C - Manufacturing
2	Large	C - Manufacturing
3	Large	C - Manufacturing
4	Large	A - Agriculture, forestry, and fishing
5	Medium	C - Manufacturing
6	Small	S - Other service activities
7	Small	M - Professional, scientific, and technical activities

**Figure 1.**
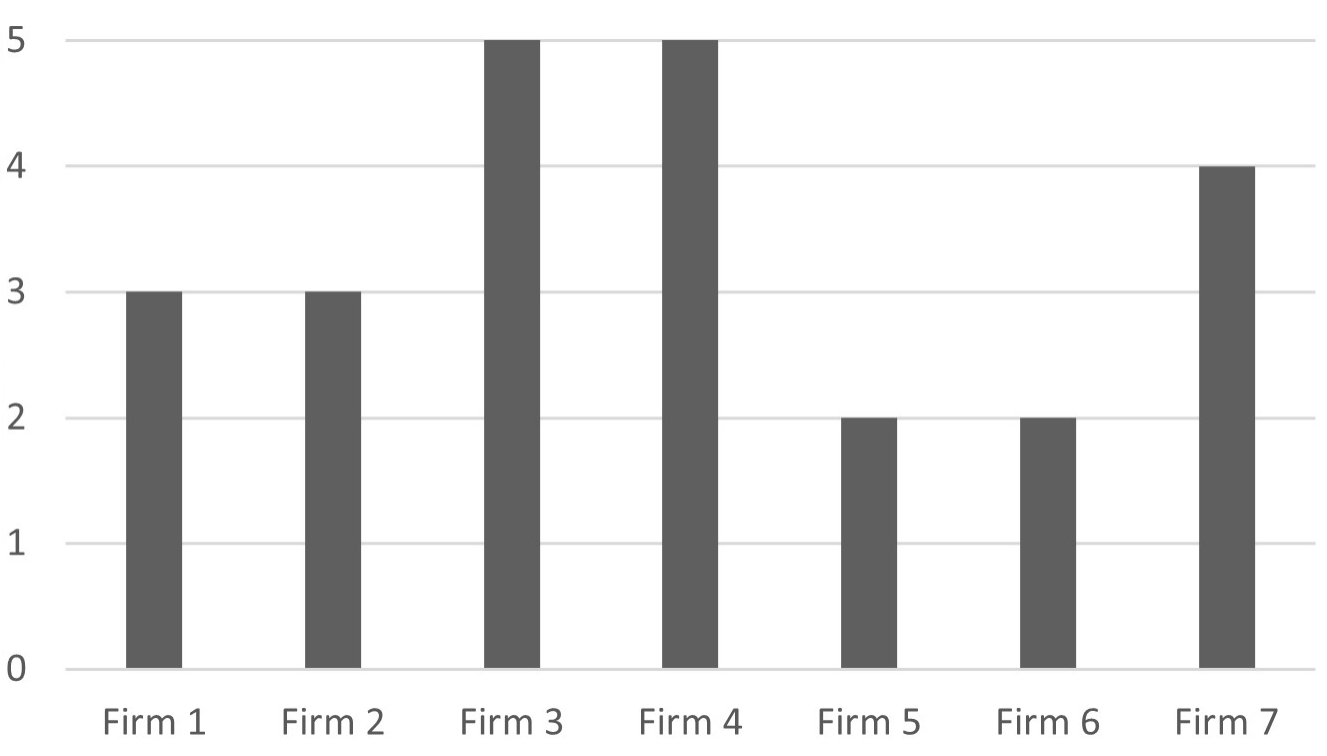
Number of I4.0 technologies used. The firms surveyed use at least two and at most five of the I4.0 technologies.

Figure 1 shows that the firms surveyed use at least two and at most five of the nine I4.0 technologies; bigger firms generally use more I4.0 technologies than smaller ones, except for Firm 7.

**Figure 2.**
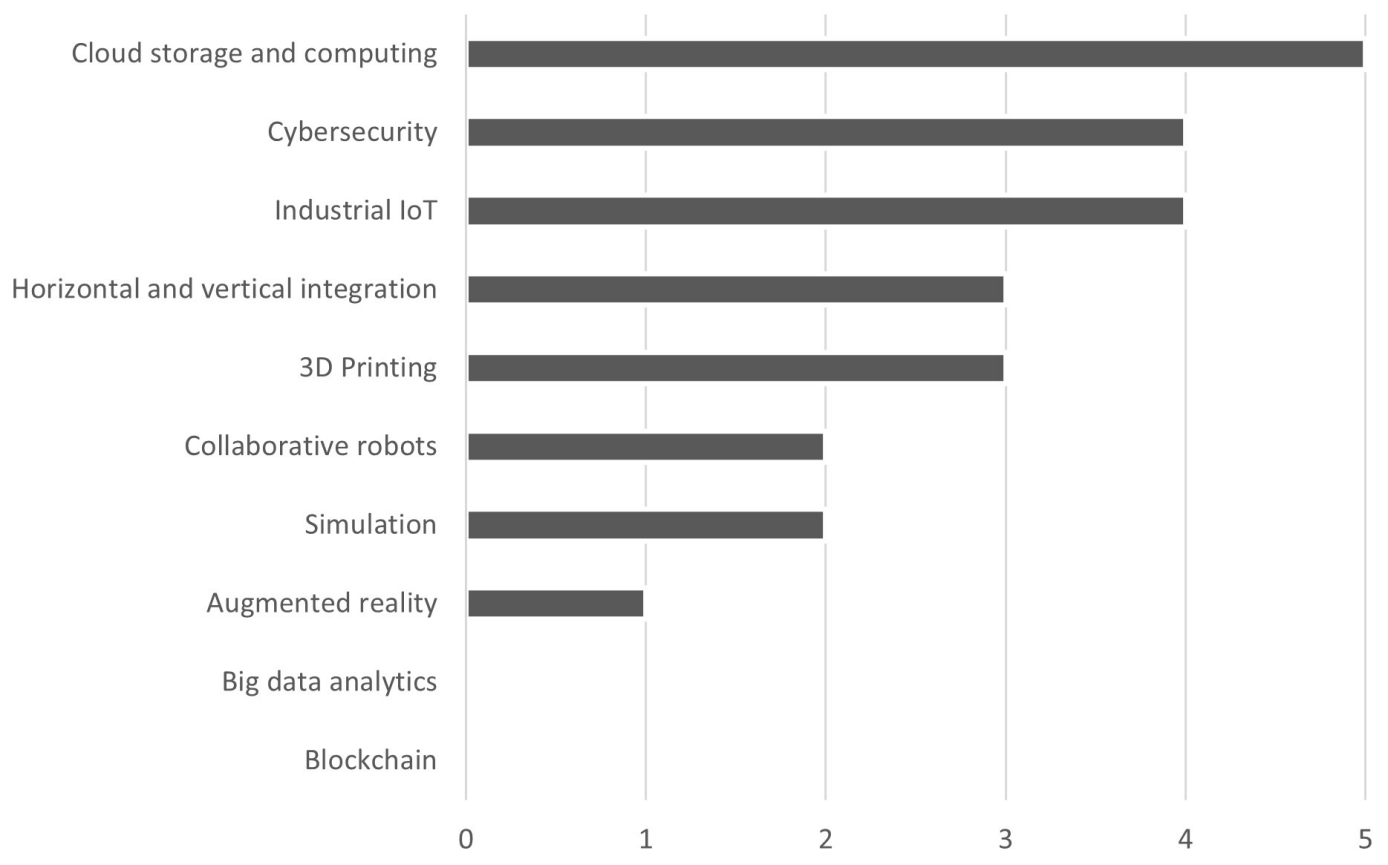
Most used I4.0 technologies. The firms use especially cloud storage and computing, cybersecurity, and industrial IoT, followed by horizontal and vertical integration and 3D printing, and finally collaborative robots, simulation, and augmented reality. None uses big data analytics, i.e., techniques for managing large amounts of data through open systems that allow forecasts or predictions, and blockchain technology.

As seen in Figure 2, the firms use especially cloud storage and computing, cybersecurity, and industrial IoT, followed by horizontal and vertical integration and 3D printing, and finally collaborative robots, simulation, and augmented reality. None uses big data analytics, i.e., techniques for managing large amounts of data through open systems that allow forecasts or predictions. All the firms surveyed declared knowing BCT but none of them uses it. Only one firm among those surveyed, which is large-sized, has analysed the potential use of BCT in its business processes, specifically for supply chain traceability.

The firms surveyed collaborate with different kinds of actors from the Marche Region, other Italian Regions, and other countries. As seen in Table 3 small-sized firms report having more collaborations than larger firms.

**Table 3.** Collaborations by type of collaborator and territorial level. 1= in the Marche Region, 2 = in other Italian regions, 3 = in other countries. Small firms report a higher number of collaborations than larger firms.

Firm	Size	Academia	Industry Clusters	Governments	DIHs	Trade ass.	Firms using I4.0	Firms providing I4.0
1	Large	1, 2	1, 2	-	-	1	-	1, 2, 3
2	Large	1	1	1, 2	1, 2	1	1, 2	1, 2
3	Large	1, 2	3	2, 3	-	2	-	-
4	Large	1, 2	-	1, 2	-	-	-	1, 2
5	Medium	-	-	-	-	-	1	1, 2
6	Small	1	1, 2	1, 2, 3	1, 2	1, 2	1, 2	1, 2
7	Small	1, 2, 3	1, 2	1, 2, 3	1	1, 2	1, 2, 3	1, 2, 3

**Figure 3.**
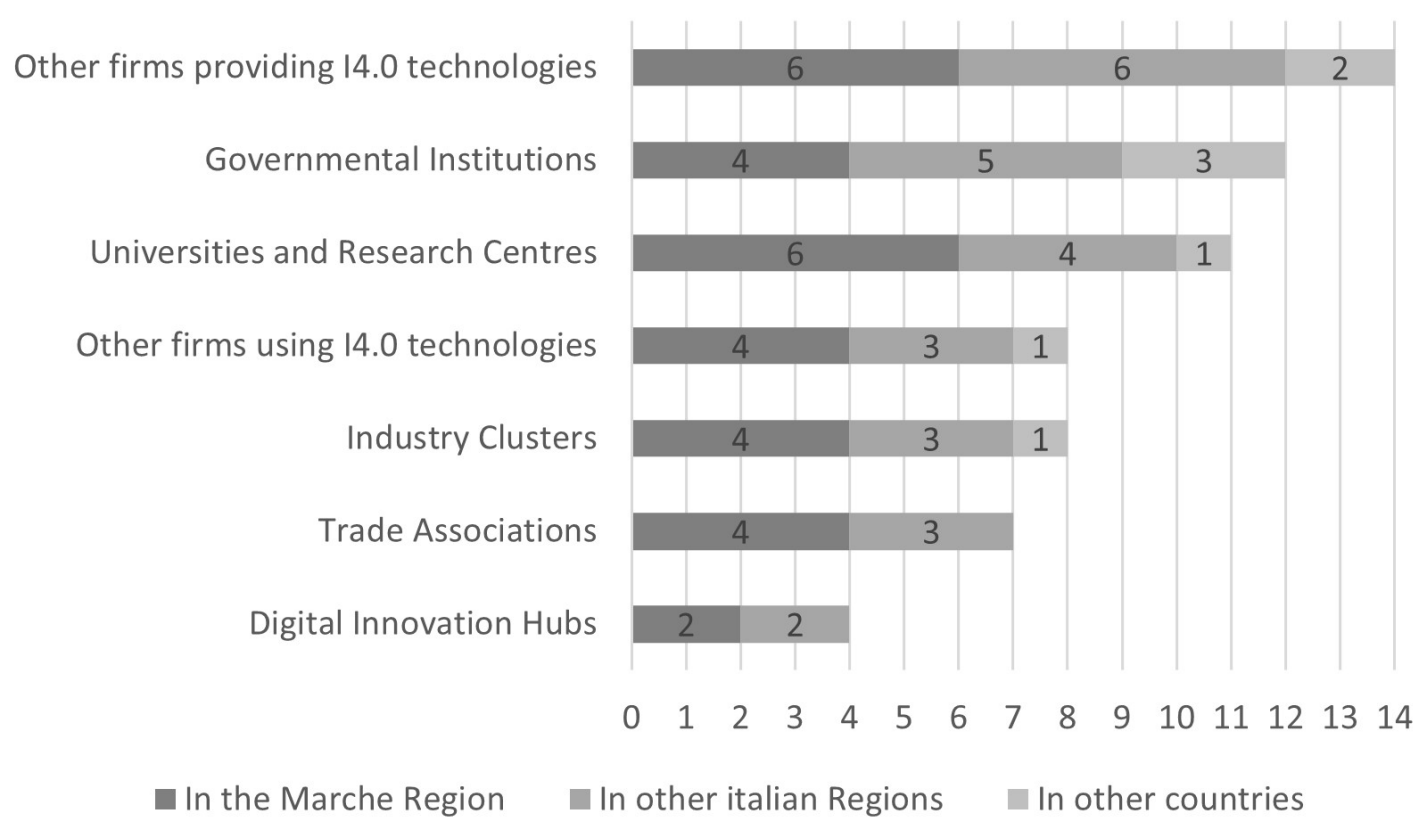
Total number of collaborations by kind of collaborator and territorial level. Most collaborations are concentrated at the regional and national levels, while collaborations in other countries are marginal. A higher number of collaborations are with providers of I4.0 technologies, governmental institutions, and universities and research centres. Lower levels of collaboration are with other firms using I4.0 technologies, Industry Clusters, and Trade Associations. Collaborations with Digital Innovation Hubs are the least present and there is an absence of collaborations with trade associations and DIHs located in other countries.

The number of collaborations was counted per type of collaboration ( Figure 3): most are concentrated at the regional (30 collaborations) and national (26) levels, while collaborations in other countries are marginal (eight). A higher number of collaborations are with providers of I4.0 technologies (14), governmental institutions (12), and universities and research centres (11). Lower levels of collaboration are with other firms using I4.0 technologies (eight), Industry Clusters (eight), and Trade Associations (seven). Collaborations with Digital Innovation Hubs are the least present (four) and there is an absence of collaborations with trade associations and DIHs located in other countries. The firms reported no other kind of collaboration in addition to those presented in the answers.

**Figure 4.**
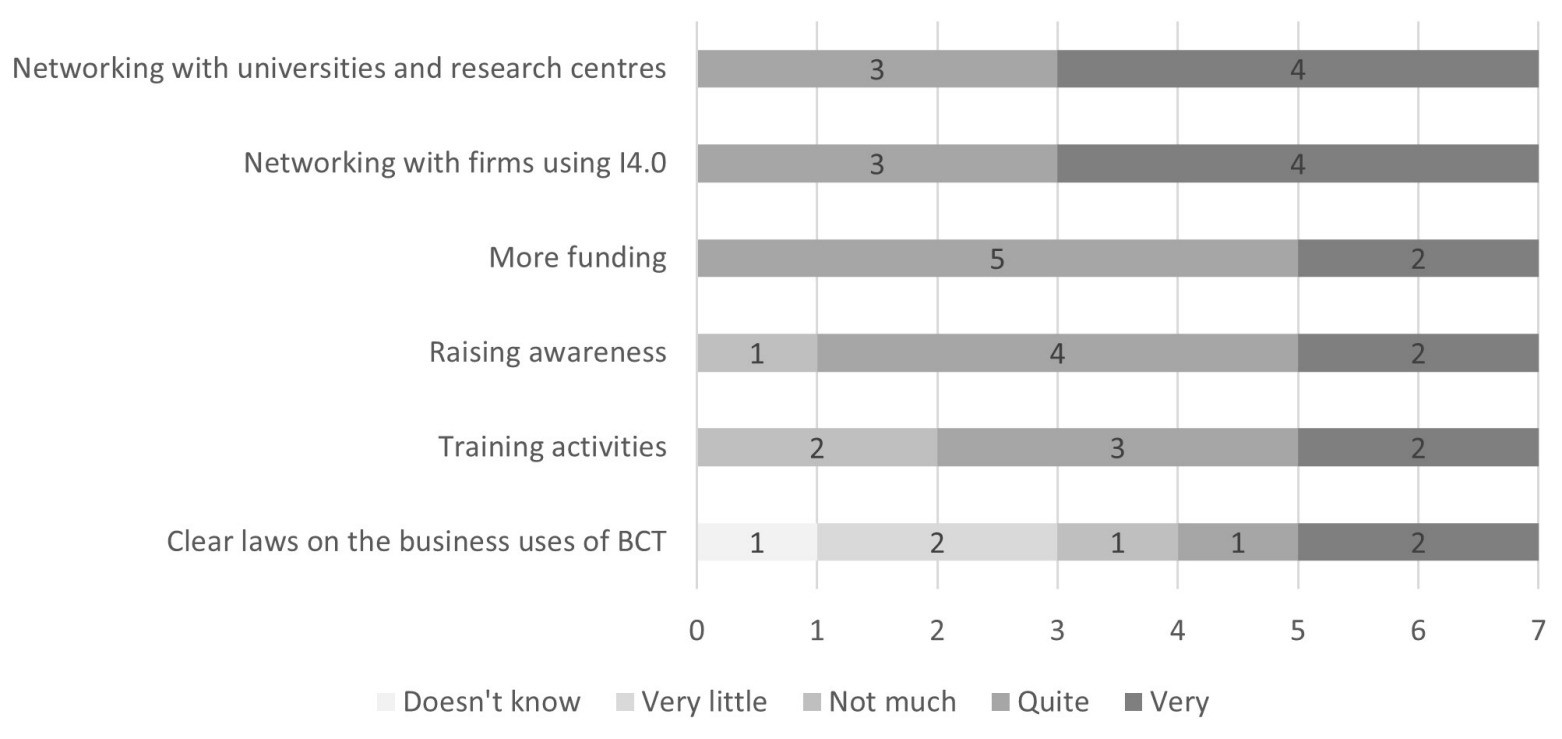
Perceived usefulness of measures for the diffusion of I4.0 technologies including BCT. Networking activities both with academia and other firms using I4.0 technologies were considered the most useful by the firms surveyed. Receiving more funding for the implementation of I4.0 technologies in firms was rated as quite useful by five firms out of seven, and very useful by the remaining two. Raising awareness about I4.0 technologies and training activities on them were rated as less useful overall but still of relevant importance. The firms gave mixed ratings to the usefulness of clear laws on the business uses of BCT for its diffusion.

The firms surveyed were asked to rate the importance of some measures for increasing the diffusion of enabling I4.0 technologies, including BCT, among firms in the Marche Region ( Figure 4). Networking activities both with academia and other firms using I4.0 technologies were considered the most useful. Receiving more funding for the implementation of I4.0 technologies in firms was rated as quite useful by five firms out of seven, and very useful by the remaining two. Raising awareness about I4.0 technologies and training activities on them were rated as less useful overall but still of relevant importance. Lastly, given the lack of clear regulations on BCT, the firms rated the usefulness of clear laws on the business uses of BCT for its diffusion. The answers were mixed, with three firms considering clear regulations not useful and the other three considering them useful, while one firm did not know what to answer. The firm which was exploring the application of BCT in its business processes considered clear regulations to be very useful. The firms were also given the possibility to suggest additional measures: firm number two proposed ‘common projects’ and firm number three added ‘Skills development in young people’ ^54^.

## Discussion

The qualitative analysis of the economic context based on scientific papers and reports showed that firms in the Marche Region struggle to adopt I4.0 technologies. Factors hindering the implementation of these innovative technologies are the small size of firms and the lack of technical knowledge and collaborations with technology providers. However, the small firms interviewed for this study use I4.0 technologies as much as the bigger firms do. Indeed, as the 2021 report of the Osservatorio Impresa 4.0 stated, the limited size of firms is not an insurmountable obstacle to the implementation of I4.0 technologies ^61^. Instead, collaborations are a driver for the use of I4.0: the firms surveyed for this study collaborate especially with other firms providing I4.0 technologies, leveraging on the providers’ technical expertise to use these innovative technologies. Moreover, they have a high number of collaborations with universities and research centres, confirming their role as knowledge and innovation generators ^41^, and with governmental institutions, which confirms the importance of government support to firms for innovation ^43, 44^. This collaboration between firms, universities, and government may indicate the existence of a TH approach that allows the firms surveyed to be innovative. Although the collaborations are mostly with universities and governments in the Marche Region and other Italian regions, international collaborations are present too, confirming the importance of international ties and networking for innovation.

The firms surveyed also reported having strong ties with other firms using I4.0 technologies, industry clusters, and trade unions, showing the importance of being part of networks of actors with similar objectives and needs. However, in this case, the collaborations were mainly with Italian actors, which may indicate a lack of interest in, knowledge of, or access to the possibility to collaborate with firms, industry clusters, and trade unions in other countries. Moreover, it is surprising that the firms interviewed do not collaborate much with Italian DIHs and at all with European DIHs, since these are knowledge brokers that help firms implement innovative technologies and intermediaries that help them create collaborations with governments, academia, and other firms.

As for the usefulness of different measures for the local diffusion of I4.0 technologies, the firms interviewed considered very or quite useful the networking activities with other firms using I4.0 technologies and with academia, and they underlined the importance of government support with funding opportunities. This reinforces the validity of a TH approach for the diffusion of I4.0 technologies in the Marche Region, promoting collaborations and networking, indeed, one of the surveyees mentioned the need for collaboration in projects on I4.0. Raising awareness and training activities were found just slightly less useful. These activities are conducted not only by universities and governments but also by trade unions and industrial clusters, with which the firms surveyed collaborate. Again, it is notable that firms do not collaborate much with Italian DIHs and at all with European DIHs, whose activities supporting firms involve raising awareness and training on innovative technologies.

Finally, BCT has been addressed in the literature as the new pillar of I4.0 technologies ^3^. The firms interviewed know BCT but only one of them studied its concrete applications to its business processes. This may be related to the novelty that this technology represents for Italian firms ^22^. Firms were asked in the questionnaire to rate the usefulness of having clear regulations on BCT, of which the absence has been found as a barrier to its adoption in firms ^36, 37^. The responses were mixed, however, the firm which had studied the implementation of BCT in its business processes considered clear regulations to be very important.

**Figure 5.**
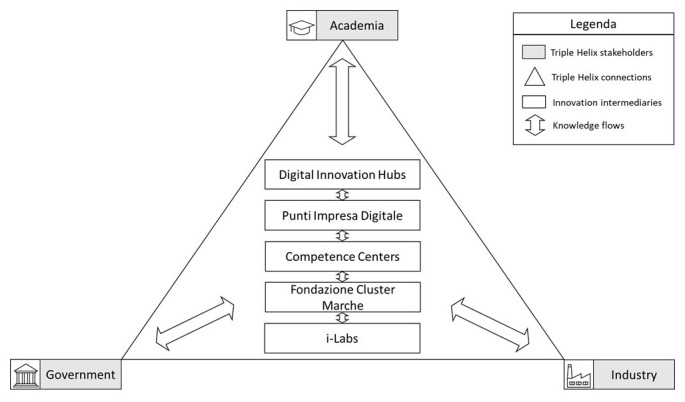
A TH model for the diffusion of I4.0 in firms in the Marche Region. Innovation intermediaries in the Marche Region such as DIHs, PID, Competence Centers, Fondazione Cluster Marche, and i-Labs are placed at the centre of the TH: they share knowledge with each other and transfer to and receive knowledge from the typical stakeholders involved in the regional TH, and intermediate the knowledge flow between these actors through collaborations which could be leveraged to spread the diffusion of I4.0 technologies in firms in the Marche Region. Source: own elaboration.

This research was driven by the limited implementation of I4.0 technologies in firms in the Marche Region and thereby aims to suggest measures to promote the adoption of these technologies, including BCT, in the regional context. The findings suggest that a TH approach could be useful to foster the diffusion of I4.0 technologies in firms in the Marche Region, leveraging on local actors. Figure 5 shows how the TH model would work in the Marche Region. Innovation intermediaries such as DIHs, PID, Competence Centers, Fondazione Cluster Marche, and i-Labs are placed at the centre of the TH. These not only share knowledge with each other but also transfer to and receive knowledge from the typical stakeholders involved in the regional TH, namely academia, government, and industry, and intermediate the knowledge flow between these actors through collaborations which could be leveraged to spread the diffusion of I4.0 technologies in firms in the Marche Region.

It identifies institutions in its territory that may be part of a TH to support the diffusion of these innovative technologies. The TH model proposed is based on both an analysis of the economic context in the Marche Region and empirical evidence from a survey of seven innovative local firms that use I4.0 technologies. The survey was conducted to collect these firms’ opinions on what other firms may need to effectively adopt these technologies.

Based on the results of the study, some measures to increase the diffusion of I4.0 in local firms can be suggested. In the perspective of raising awareness on I4.0 technologies, policymakers may want to continue fostering a TH approach by helping strengthen the collaboration between some key institutions in the Marche Region: Punto Impresa Digitale (PID), Digital Innovation Hubs (DIHs), Competence Centers, Fondazione Cluster Marche, and i-Labs. These could have the role of innovation intermediaries, i.e., institutions that connect firms with governmental institutions and academia in the regional TH.

The survey evidenced a lack of collaboration with DIHs, despite their role as connectors among the three helices. DIHs can help firms with the practical implementation of I4.0 technologies and regional institutions by adjusting financial and training incentives to the needs of different firms. Additionally, DIHs can connect firms and institutions with other intermediaries around Europe to facilitate international access and exchange of knowledge, allowing them to gather experiences and best practices from other European contexts on the implementation of I4.0 technologies. Both policymakers and DIHs need to make firms more aware of the possibility to use DIHs to access knowledge from all around Europe. On one hand, policymakers need to reinforce the role of existing DIHs and give them more visibility with entrepreneurs. On the other hand, DIHs should organize events with firms and institutions of different European countries that already use Industry 4.0 technologies and BCT and are willing to share their direct experience, so that firms in the Marche Region can appreciate the value that DIHs can provide them.

As for the policies, an important role in the implementation of a TH for the diffusion of I4.0 has been individuated in the S3. The involvement of the key intermediaries in the Marche Region in the S3 must be fostered by policymakers to facilitate its implementation.

However, caution is mandatory before generalising this study’s results. Indeed, although the seven surveyed firms’ expertise, based on the practical implementation and use of I4.0 technologies, is relevant to the objective of this study, their limited number and high level of digitalization make them not representative of all the firms in the Marche Region.

Finally, some research suggestions can be proposed. This research addressed the importance of the lack of clear legislation on BCT, on which the interviewees’ responses were mixed, thus inconclusive, and cannot be translated into recommendations for policymakers. However, they constitute a relevant topic for future research: the lack of consensus among firms about the usefulness of clear regulations on BCT needs to be further investigated, to understand if it is due to a lack of awareness of the legal implications of using BCT or other reasons. Furthermore, it would be of interest to develop multiple case studies to understand more in-depth if and how the TH influenced the adoption of I4.0 technologies in regional firms. Finally, it would be useful to collect insights from TH stakeholders and intermediaries to identify opportunities and challenges in collaborating with firms on I4.0 and specifically BCT.

## Data Availability

Zenodo: Survey I4.0 in the Marche Region. https://doi.org/10.5281/zenodo.7561154 ^55^. This project contains the following underlying data: Survey I4.0 in the Marche Region.csv. (Anonymised Italian responses to the survey from seven firms). Zenodo: Survey I4.0 in the Marche Region (ENG translation). https://doi.org/10.5281/zenodo.7728353 ^54^. This project contains the following underlying data: Survey I4.0 in the Marche Region (ENG translation).csv (English translation of the anonymised Italian responses to the survey from seven firms). Zenodo: Questionario - Le tecnologie abilitanti nelle Marche. https://doi.org/10.5281/zenodo.7695303 ^53^. This project contains the following extended data: Questionario - Le tecnologie abilitanti nelle Marche.pdf. (PDF print of the original questionnaire in Italian). Zenodo: Questionnaire for a survey on Industry 4.0 in firms of the Marche Region, Italy. https://doi.org/10.5281/zenodo.7695298 ^52^. This project contains the following extended data: Questionnaire - Enabling technologies in the Marche Region.pdf. (PDF print of the questionnaire translated in English from Italian). Data are available under the terms of the Creative Commons Attribution 4.0 International license (CC-BY 4.0).
