# Supplementary material for: A triple helix model for the diffusion of Industry 4.0 technologies in firms in the Marche Region
Source: Open Res Eur. 2023 Nov 23;3:89. Originally published 2023 Jun 9. [Version 2] doi: 10.12688/openreseurope.15706.2 (PMC10774631; doi:10.12688/openreseurope.15706.2)
Supplement: Peer review history

# Reviewer report: Reviewer response for version 2

**DOI:** 10.21956/openreseurope.18228.r36408

**Published:** 2024-01-08

**Authors:** Andrea Ferrannini

**Affiliations:** 1 Department of Economics and Management, Universita degli Studi di Firenze, Florence, Tuscany, Italy

Dear Author,

 thanks for having submitted a revised manuscript. 

 The manuscript has truly improved following all suggestions.

If applicable, is the statistical analysis and its interpretation appropriate?

Not applicable

Is the study design appropriate and is the work technically sound?

Partly

Is the work clearly and accurately presented and does it engage with the current literature?

Partly

Are the conclusions drawn adequately supported by the results?

Yes

Are sufficient details of methods and analysis provided to allow replication by others?

Yes

Are all the source data and materials underlying the results available?

Yes

Reviewer Expertise:

Industrial policy; Local economic development; Sustainability transition; SDG localization

I confirm that I have read this submission and believe that I have an appropriate level of expertise to confirm that it is of an acceptable scientific standard.

# Reviewer report: Reviewer response for version 2

**DOI:** 10.21956/openreseurope.18228.r36407

**Published:** 2023-11-27

**Authors:** Petrus Letaba

**Affiliations:** 1 University of Pretoria, Pretoria, Gauteng, South Africa

I am satisfied with the changes effected.

If applicable, is the statistical analysis and its interpretation appropriate?

Partly

Is the study design appropriate and is the work technically sound?

Partly

Is the work clearly and accurately presented and does it engage with the current literature?

Partly

Are the conclusions drawn adequately supported by the results?

No

Are sufficient details of methods and analysis provided to allow replication by others?

Yes

Are all the source data and materials underlying the results available?

Yes

Reviewer Expertise:

Technology roadmapping, technology foresighting, technology assessment, technology adoption, sociotechnical transitions, etc.

I confirm that I have read this submission and believe that I have an appropriate level of expertise to confirm that it is of an acceptable scientific standard.

# Reviewer report: Reviewer response for version 1

**DOI:** 10.21956/openreseurope.16969.r33924

**Published:** 2023-08-14

**Authors:** Andrea Ferrannini

**Affiliations:** 1 Department of Economics and Management, Universita degli Studi di Firenze, Florence, Tuscany, Italy

The study engages with very relevant and vibrant debates – industry 4.0 and triple helix – and applies both framework to the case-study of Marche Region’s context. It adopts multiple methods to provide new evidence and build a triple helix model is proposed to foster the diffusion of Industry 4.0 technologies.

 To be fully accepted for indexing, I would suggest the following improvements.

1. In the introduction, to provide more details about the economy of Marche Region and its structure, thus explaining to the reader its relevance.
2. In the theoretical framework, to engage more in depth with both Industry 4.0 literature and TH literature – if the max length of the article allows it. In particular, it is suggested to engaging with the debate on intermediary institutions – as this is the main conclusion of the study. Among others, see this paper and the cited references: Prodi *et al. *2022 ^1^
3. In the study design, to mention other previous studies that used a similar design and methods, thus better justifying its acceptance in the current debate and reliability.
4. In the study design, to explain why the survey was open only for three days and if any previous contact with firms members of the members of the Fondazione Cluster Marche was done. In addition, to explain if – and to what extent – these 7 firms may be illustrative at least of a niche of firms in the Marche Region.
5. In the study design, to explain if and what other data collection tools were excluded – e.g., focus group discussion with local innovative firms; semi-structured interviews with key informants, etc.  
6. In the discussion, to better explain what is new in your paper and what confirms previous research in the topic and in the region, along with discussing the external validity (if any) of the results.

If applicable, is the statistical analysis and its interpretation appropriate?

Not applicable

Is the study design appropriate and is the work technically sound?

Partly

Is the work clearly and accurately presented and does it engage with the current literature?

Partly

Are the conclusions drawn adequately supported by the results?

Yes

Are sufficient details of methods and analysis provided to allow replication by others?

Yes

Are all the source data and materials underlying the results available?

Yes

Reviewer Expertise:

Industrial policy; Local economic development; Sustainability transition; SDG localization

I confirm that I have read this submission and believe that I have an appropriate level of expertise to confirm that it is of an acceptable scientific standard, however I have significant reservations, as outlined above.

## References

[1] : Industry 4.0 policy from a sociotechnical perspective: The case of German competence centres. *Technological Forecasting and Social Change* .2022;175: 10.1016/j.techfore.2021.121341 10.1016/j.techfore.2021.121341

# Reviewer report: Reviewer response for version 1

**DOI:** 10.21956/openreseurope.16969.r33980

**Published:** 2023-08-07

**Authors:** Petrus Letaba

**Affiliations:** 1 University of Pretoria, Pretoria, Gauteng, South Africa

This article confirms what is known that the Triple Helix (TH) collaborations between business, government and academia have a potential to increase the innovation potential of a region (Mache Region in Italy is used as a case study in this paper). A specific innovation that is at the centre of focus for this paper is that related to the Fourth Industrial Revolution focusing on the sectors of manufacturing, services as well as agriculture, forestry and fishing. The unique contribution of this paper within the TH body of knowledge is identification of the innovation intermediaries that are placed at the centre of TH triangle to facilitate knowledge flow and transfer between the actors. The important role of TH intermediaries was observed by Letaba (2019).

 The methodology used is robust and reasonable, with a sufficient triangulation (systematic literature review, documents analysis and qualitative survey). One observation on improvement of the methodology is that only Scopus database was used although it is mostly recommended to use at least two databases. As there is a significant overlap between Scopus and another important database such as Web of Science, the impact is not so huge, but few useful papers can be left out.

 The authors were requested to include a Conclusion section and to correct some minor grammatical errors that are in the manuscript.

If applicable, is the statistical analysis and its interpretation appropriate?

Partly

Is the study design appropriate and is the work technically sound?

Partly

Is the work clearly and accurately presented and does it engage with the current literature?

Partly

Are the conclusions drawn adequately supported by the results?

No

Are sufficient details of methods and analysis provided to allow replication by others?

Yes

Are all the source data and materials underlying the results available?

Yes

Reviewer Expertise:

Technology roadmapping, technology foresighting, technology assessment, technology adoption, sociotechnical transitions, etc.

I confirm that I have read this submission and believe that I have an appropriate level of expertise to confirm that it is of an acceptable scientific standard, however I have significant reservations, as outlined above.

## References

[1] : Relationship between Dominant Triple Helix Model and Type of Intermediary Organizations. *2019 Portland International Conference on Management of Engineering and Technology (PICMET), Portland, OR, USA* .2019;1-6
